# Estrogen Receptor and Claudin-6 Might Play Vital Roles for Long-Term Prognosis in Patients With Luminal A Breast Cancer Who Underwent Neoadjuvant Chemotherapy

**DOI:** 10.3389/fonc.2022.630065

**Published:** 2022-06-30

**Authors:** Yushi Liu, Ye Kang, Jianyi Li, Yang Zhang, Shi Jia, Qiang Sun, Yan Ma, Jing Zhang, Zhenrong Wang, Yanan Cao, Yang Shen

**Affiliations:** ^1^Department of Breast Surgery, ShengJing Hospital of China Medical University, Shenyang, China; ^2^Department of Pathology, Shengjing Hospital of China Medical University, Shenyang, China; ^3^Department of Breast Surgery, Liaoning Cancer Hospital, Shenyang, China; ^4^Department of Breast Surgery, Benxi Iron and Steel Co. General Hospital, Benxi, China

**Keywords:** luminal A breast cancer, cycling hypoxia, estrogen receptor, claudin-6, neoadjuvant chemotherapy

## Abstract

**Purpose:**

It is well-known that the pathological complete response (pCR) rate in patients with luminal A cancer (LAC) is lower than those of other subtypes of breast cancer. The phenotype of cancer often alters after neoadjuvant chemotherapy (NAC) which may be related to hypoxia, and the latter might induce the drift of the estrogen receptor (ER). The phenotype drift in local advanced LAC after NAC might influence the long-term prognosis.

**Methods:**

The oxygen concentration of cancer tissues during NAC was recorded and analyzed (n = 43). The expression of ER and claudin-6 was detected in pre- and post-NAC specimens.

**Results:**

NAC might induce the cycling intracanceral hypoxia, and the pattern was related to NAC response. The median follow-up time was 61 months. Most of the patients (67%) with stable or increased ER and claudin-6 expression exhibited perfect prognosis (DFS = 100%, 61 months). About 20% of patients with decreased claudin-6 would undergo the poor prognosis (DFS = 22.2%, 61 months). The contrasting prognosis (100% vs. 22.2%) had nothing to do with the response of NAC in the above patients. Only 13% patients had stable claudin-6 and decreased ER, whose prognosis might relate to the response of NAC.

**Conclusion:**

NAC might induce cycling intracanceral hypoxia to promote the phenotype drift in local advanced LAC, and the changes in ER and claudin-6 after NAC would determine the long-term prognosis.

## Introduction

Neoadjuvant chemotherapy (NAC) is the standard treatment for patients with locally advanced breast cancer (LABC) or big cancers seeking breast-conserving surgery (BCS) (1). The St. Gallen subtype classification, introduced in 2011, categorizes breast cancer into luminal A, luminal B HER2-neu negative, luminal B HER2-neu positive, HER2-neu non-luminal, and triple negative breast cancer (TNBC) based on immunohistochemical staining (2). For patients with TNBC or HER2 overexpression (OE), achieving a pathological complete response (pCR) to NAC may predict favorable outcomes (3). However, in breast cancer patients with a luminal type, the response to NAC cannot predict the outcomes (4). Furthermore, compared with patients with TNBC or HER2 OE, patients with luminal A cancer (LAC) have the lowest pCR rate, but the prognosis is generally better (5, 6). This suggests a complicated expression in the NAC process for LAC patients, and there may be a considerable proportion of patients who both exhibit chemoresistance and maintain a good prognosis. Therefore, there is an urgent need for sensitive clinical indicators to predict the prognosis of patients with LAC who would undergo NAC.

It is reported that there is a relationship between intracanceral hypoxia and chemoresistance (7). A recent study using tomographic near-infrared diffuse optical spectroscopy demonstrated decreased oxygenation in cancer tissue in patients with effective NAC, while the oxygenation in patients with drug resistance remained unchanged (8). Hypoxia induces a poor therapeutic response and outcome in breast cancer, especially in estrogen receptor (ER)-positive patients (9). Meanwhile, under hypoxia, the increased expression of HIF-1α might cause the loss of the ER protein (10). ER-positive breast cancer cells may evade treatment by entering dormancy, which leads to a poor response (11). On the other hand, changes in ER, progesterone receptor (PR), HER2, and Ki67 status after NAC are common in breast cancer (11–13). These findings suggested that breast cancer tissue of the same patient has temporal heterogeneity during NAC (14). The change in the ER status after NAC may be associated with chemoresistance and prognosis.

Claudin-6 (CLDN6), a member of the claudin transmembrane protein family, plays an important role in regulating paracellular permeability and maintaining cell polarity in epithelial and endothelial cell sheets (15). HIF-1α accumulation under hypoxia might promote CLDN6 transcription, and increased CLDN6 would weaken HIF-1α protein stability and slow down hypoxia-induced breast cancer metastasis (16). Osanai et al. found that a decreased expression of CLDN6 may promote the formation of breast cancer, suggesting that CLDN6 may act as a cancer suppressor, and its downregulation may contribute to the malignant progression of luminal breast cancers (17). CLDN6 downregulation contributes to enhance the cancerigenic and invasive properties of LAC cells (15). A recent study found that the expression of CLDN6 may be induced by ERα (18).

In our present study, the oxygen concentrations of cancer tissues with LABC were monitored during NAC; the expressions of ER and CLDN6 before and after NAC were detected; and the relationship between the NAC response and the expressions of ER and CLDN6 was analyzed. The survival analysis was also conducted on the long-term follow-up data, and NAC reactivity and changes in the expressions of ER and CLDN6 were used to predict the long-term prognosis.

## Materials and Methods

### Oxygenation of Cancer Tissue Associated With NAC

Between June 1, 2014, and May 31, 2015, a total of 43 patients with LAC and positive nodes who were treated with biopsy, NAC, surgery, and systemic treatment were recruited in the Department of Breast Surgery at the Shengjing Hospital of China Medical University, Shenyang, China. The inclusion criteria were (1) no prior history of breast cancer or other malignancies; (2) not pregnant and lactating during diagnosis; (3) invasive ductal carcinoma diagnosed by biopsy; and (4) ER expressions ≥20% and Ki67 <20% by IHC before NAC. Exclusion criteria were (1) the skin or chest wall invaded by the cancer; (2) connective tissue disease and dermatosis; and (3) metastatic breast cancer. According to RECIST 1.1 (19), patients were categorized into four subgroups based on their responses to NAC: CR, PR, SD, PD.

**Figure 1:**
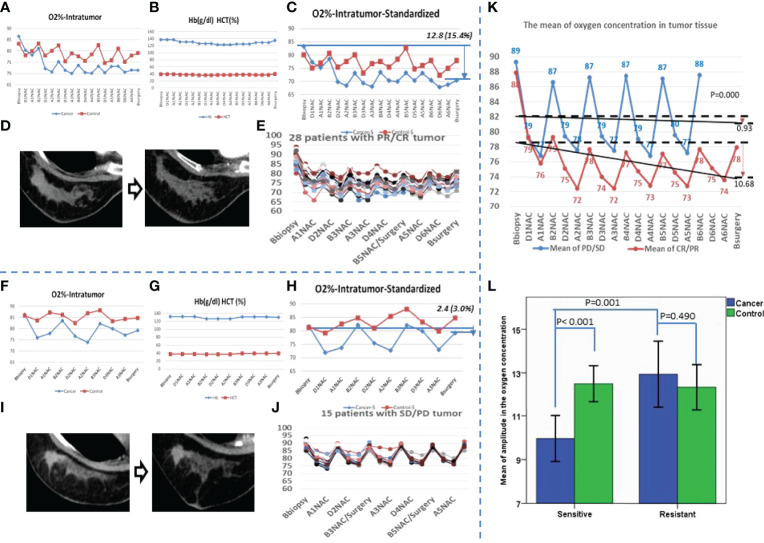
The variation curve of SpO_2_ of the patients who were sensitive or resistant for the neoadjuvant chemotherapy. **(A)** A patient with partial response (PR) cancer after neoadjuvant chemotherapy (NAC) for 6 cycles. The O_2_% of both affected breast and the contralateral breast are shown; the red line shows the contralateral breast, and the blue line shows the affected breast. **(B)** The hemoglobin (Hb) and hematocrit (HCT) were recorded at the same time for the patient in photo **(A, C)** The standardized result of the patient in photo A corrected by Hb and HCT are shown; the red line shows the contralateral breast, and the blue line shows the affected breast. **(D)** The cancer imaging of the patient in photo A by contrast enhancement computed tomography (CECT) before NAC and after NAC. **(E)** After NAC, 28 patients’ cancer was sensitive, and the oxygen concentration of cancer tissue before and after NAC. **(F)** A patient with stable disease (SD) cancer after NAC for 3 cycles; the O_2_% of both affected breast and contralateral breast are shown; the red line shows the contralateral breast, and the blue line shows the affected breast. **(G)** The Hb and HCT were recorded at the same time for the patient in photo **(F, H)** The standardized result of the patient in photo F corrected by the Hb and HCT; the red line shows the contralateral breast, and the blue line shows the affected breast. **(I)** The cancer imaging of the patient in photo F by CECT before NAC and after NAC. **(J)** After NAC, 15 patients’ cancer was resistant; the oxygen concentration of cancer tissue before and after the NAC is also shown. **(K)** The blue line shows the mean oxygen concentration of every checkpoint for patients with resistant cancer, the red line shows the sensitive group, and the black lines show the trends of the mean oxygen concentration. **(L)** The mean of amplitude in oxygen concentration is presented. Blue shows the affected breast, and green shows the contralateral breast.

For each participant, cancer tissue oxygenation was measured using the Near-infrared Oxygenation Detector (Foresight, P/N 01-06-2030C, CasMed) before biopsy, 24 h before and after each cycle of NAC, during each cycle of NAC, and before surgery $\text{[standardized result of intracanceral oxygenationconcentration = }intratumoral\;O2\%\times \left(\frac{Hb\;of\;each\;check\;point/HCT\;of\;each\;check\;point\;}{Hb\;before\;NAC/HCT\;before\;NAC}\times 100\%\right)]$ (**Figure 1**). All the participants agreed to undergo magnetic resonance image (MRI) and contrast-enhanced computed tomography (CT) to evaluate the chemotherapy response. Anthropometric data (age at diagnosis, menstrual history, family history, surgery, chemotherapy, and endocrine therapy) as well as cancer-related variables (size, location, histological grade, cancer thrombosis, nodes, ER expressions by IHC before and after NAC) were collected.

### Immunohistochemistry

IHC detecting ER, PR, HER2, Ki67, and CLDN6 was performed on formalin-fixed, paraffin-embedded cancer samples obtained from the Department of Histology and Pathology of the hospital. The details of the operation process were consistent with the previous study (20). Ki67 status was expressed as a percentage of positive cells with a threshold of 20% of positive cells (21). Based on the St. Gallen Consensus 2013, luminal A breast cancer should be matched with ER ≥20% and/or PR ≥20%, HER2 negative, and Ki67 <20% (22). The pathological process and the antibodies used in the samples of both biopsy before NAC and surgery after NAC remained the same. The ER phenotype drift was determined by the expression change of ER after NAC which was more than 10% compared with that before NAC. According to the ER changes, patients were allocated into three subgroups: ER increased (range of ER rise >10% after NAC), ER stable (range of ER change ≤10% after NAC), and ER decreased (range of ER descend >10% after NAC).

All specimens were stained with 1:500 diluted anti-CLDN6 antibody (ab107059, Abcam, Cambridge, UK). Pathological results were evaluated by two pathologists who were blind to the protocol. For CLDN6, each tissue sample was scored according to its staining intensity (0, none; 1, weak; 2, moderate; 3, strong) multiplied by the point of the percentage of stained cells (0: positive cells = 0; 1: positive cells ≤25%; 2: 26%–50%; 3: 51%–75%; 4: ≥75%) (23). The range of this calculation was 0–12. The CLDN6 drift criterion was that the absolute change of the CLDN6 percentage $\left(\text{change in CLDN}6\text{ expression}=\frac{the\;score\;before\;NAC-the\;score\;after\;NAC}{the\;score\;before\;NAC}\times 100\%\right)$ was >10%. According to the change in CLDN6, patients were allocated into three subgroups: CLDN6 increased (the range of CLDN6 rise >10% after NAC), CLDN6 stable (the range of CLDN6 change ≤10% after NAC), and CLDN6 decreased (the range of CLDN6 descend >10%).

### Clinical Retrospective Study

Participants were followed up with 3-month intervals in the first 2 years postsurgery, and with 6-month intervals thereafter until December 31, 2019. The diagnosis of local recurrence or contralateral breast cancer was supported by biopsy, and distant metastasis was diagnosed by biopsy or positron emission tomographic-computer tomography (PET-CT). Disease-free survival (DFS) was defined as the period between the first day after surgery and the date when first local recurrence or distant metastasis was confirmed. Overall survival (OS) was calculated from the first day after surgery to death or December 31, 2019. Anthropometric data and cancer-related variables were collected. The histological grades of the cancers were classified into grades I–III based on the Nottingham combined histological grade (24).

Because of the small sample sizes of CR and PD, based on their response we regrouped the patients as sensitive to NAC (patients with CR or PR cancers) and resistant to NAC (patients with SD or PD cancers). Because of the small sample size in the group of ER increased, based on the ER changes patients were regrouped as ER increased and stable group (patients with ER increased and ER stable) and ER decreased group (patients with ER decreased).

### Statistical Analysis

All statistical analyses were performed by SPSS software (version 17.0 for Windows). The differences in biological factors between groups were examined by Student *t* test, chi-square test, or rank-sum test. For the survival analysis, Kaplan–Meier curves were built for OS and DFS analyses. The log-rank test was used to compare survival differences among the groups. Cox proportional hazard models were established to calculate relative risk accounting for covariates.

### Ethical approval

According to the Declaration of Helsinki, all the participants signed informed consent. The study was approved by the Ethics Committee of Shengjing Hospital of China Medical University.

## Result

### Oxygenation of Cancer Tissue Associated With NAC

Among the 43 patients, the numbers of patients with complete remission (CR), partial remission (PR), stable disease (SD), and progressive disease (PD) cancers were 2, 26, 12, and 3, respectively (**Table 1**). Correction was made to eliminate the effect of NAC on hemoglobin and hematocrit (**Figure 1**). For CR and PR cancers, there was a decreasing trend in intracanceral oxygen concentrations with the cycle of NAC (**Figure 1E**). Conversely, oxygen concentrations remained relatively stable in patients with SD and PD cancers (**Figure 1J**). The level of the oxygen concentrations appeared to be reduced during and after each cycle of NAC but restored to the pre-NAC level before the next cycle of NAC, suggesting that cycling hypoxia might be induced by NAC (**Figures 1E, J**). The mean intracanceral oxygen concentration in the NAC sensitive group showed a downward trend, whereas the concentration remained stable in the resistant group (p < 0.001) (**Figure 1K**). The amplitude of the mean oxygen concentration in the resistant group was higher than that in the sensitive group (p < 0.001) (**Figure 1L**).

**Table 1:** Patient characteristics and survival analysis.

Characteristic	CR+PR(n = 28)	SD+PD(n = 15)	Statistics	P	ER increased and stable (n = 25)	ER decreased(n = 18)	Statistics	P	CLDN6 increased (n = 13)	CLDN6 stable(n = 21)	CLDN6 decreased (n = 9)	Statistics	P
**Age (years)**	52.18 ± 6.37	50.53 ± 7.82	0.555	0.460	51.28 ± 6.21	52.06 ± 7.85	0.131	0.719	50.62 ± 7.07	42.90 ± 6.60	50.00 ± 7.38	0.749	0.479
**Menopause**			0.073	0.788			1.156	0.289				2.530	0.092
**Postmenopausal**	18	9			14	13			5	15	7		
**Premenopausal**	10	6			11	5			8	6	2		
**Family history**			0.224	0.638			0.587	0.448				0.142	0.868
**No**	24	12			20	16			11	17	8		
**Yes**	4	3			5	2			2	4	1		
**Diameter before NAC (cm)**	10.104 ± 3.486	8.273 ± 2.161	3.409	0.072	9.17 ± 3.18	9.95 ± 3.23	0.712	0.404	9.48 ± 3.19	9.71 ± 3.47	8.88 ± 2.71	0.207	0.814
**Diameter after NAC (cm)**	4.189 ± 2.300	7.760 ± 2.443	22.558	0.000	5.14 ± 2.62	5.84 ± 3.27	0.599	0.443	4.69 ± 2.37	5.62 ± 2.82	6.09 ± 3.75	0.701	0.502
**RECIST**			0.046	0.830			0.095	0.759				1.504	0.235
**CR**					1	1			1	1	0		
**PR**					14	12			9	12	5		
**SD**					9	3			3	7	2		
**PD**					1	2			0	1	2		
**Quadrant**			0.046	0.830			0.493	0.486				0.630	0.538
**Areolar**	0	1			1	0			0	0	1		
**Inner upper**	3	2			4	2			1	3	2		
**Inner lower**	4	3			3	3			0	5	1		
**Outer lower**	4	2			6	0			4	2	0		
**Outer upper**	17	7			11	13			8	11	5		
**Histological grade after NAC**			0.265	0.610			1.364	0.250				0.194	0.824
**I**	2	1			2	1			1	2	0		
**II**	12	5			7	10			6	6	5		
**III**	14	9			16	7			6	13	4		
**Cancer thrombosis after NAC**			21.947	0.000			1.223	0.275				1.241	0.300
**Negative**	24	4			18	10			10	14	4		
**Positive**	4	11			7	8			3	7	5		
**Number of positive nodes after NAC**	2.04 ± 1.90	5.93 ± 4.64	15.289	0.000	2.48 ± 1.94	4.67 ± 4.89	4.134	0.049	1.69 ± 1.49	2.71 ± 2.51	7.44 ± 4.98	11.102	0.000
**Number of nodes**	24.50 ± 3.16	30.13 ± 4.16	22.909	0.000	26.24 ± 3.93	26.78 ± 5.37	0.144	0.706	25.46 ± 3.46	26.00 ± 4.07	29.00 ± 6.17	1.912	0.161
**ER before NAC (%)**	47.86 ± 17.29	40.00 ± 13.63	2.317	0.136	44.40 ± 16.09	46.11 ± 17.20	0.112	0.740	48.46 ± 14.05	45.71 ± 18.32	38.89 ± 14.53	0.932	0.402
**ER after NAC (%)**	43.39 ± 18.71	35.67 ± 18.31	1.690	0.201	45.12 ± 16.38	32.50 ± 21.51	6.744	0.013	48.08 ± 12.51	41.67 ± 18.86	27.78 ± 20.93	3.568	0.038
**Change on ER**			0.007	0.935								9.290	0.000
**Rise**	2	2							11	14	2		
**Stable**	18	9							2	2	0		
**Descend**	8	4							0	5	7		
**Operation**			0.167	0.685			1.945	0.171				1.052	0.359
**MRM**	25	12			20	17			10	19	8		
**BRS**	2	3			4	1			2	2	1		
**BCS**	1	0			1	0			1	0	0		
**AC program**			2.548	0.118			1.271	0.266				2.018	0.146
**None**	23	9			17	Z15			9	14	9		
**TAC**	5	6			8	3			4	7	0		
**Endocrine therapy**			81.364	0.000			0.047	0.829				0.157	0.855
**OFS+AI+fulvestrant**	0	6			5	1			3	2	1		
**AI+fulvestrant**	3	9			5	7			0	8	4		
**OFS+AI**	10	0			6	4			5	4	1		
**AI**	15	0			9	6			5	7	3		
**Overall survival**	95.4%	93.3%	0.202	0.655	100%	88.9%	2.980	0.092	100%	100%	77.8%	4.518	0.017
**Survival**	27	14			25	16			13	21	7		
**Dead**	1	1			0	2			0	0	2		
**Median survival time**	67.00	67.00	0.655	0.423	67	67	4.128	0.049	67.00	67.00	63.00	5.575	0.007
**Disease-free survival**	85.7%	66.7%	1.827	0.184	92.0%	61.1%	6.417	0.015	100%	90.5%	22.2%	17.917	0.000
**Disease-Free Survival**	24	10			23	11			13	19	2		
**Contralateral breast cancer**	2	2			1	3			0	1	3		
**Bone metastasis**	1	2			1	2			0	1	2		
**Bone and lung metastasis**	1	1			0	2			0	0	2		
**Median disease-free survival time**	67.00	66.00	1.318	0.258	67	66	6.531	0.006	67.00	67.00	26.75	23.748	0.000

### Immunohistochemistry

**Figure 2:**
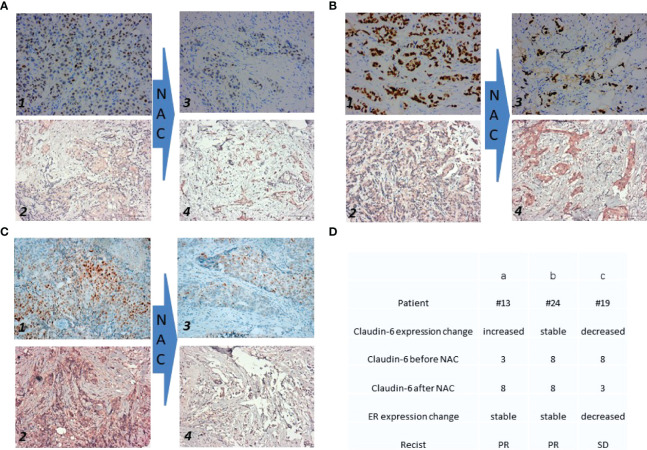
Expression of ER and Claudin-6 before and after the neoadjuvant chemotherapy. Each patient has twice immunohistochemical (IHC) results of estrogen receptor (ER) and claudin-6 (CLDN6). The expression of ER before NAC is shown on photo 1 in **(A–C)**. The expression of CLDN6 before NAC is shown on photo 2 in **(A–C)**. The expression of ER after NAC is shown on photo 3 in **(A–C)**. The expression of CLDN6 after NAC is shown on photo 4 in **(A–C)**. **(D)** The table shows the details of the three patients in **(A–C)**. For CLDN6 before and after NAC, each tissue sample was scored according to its staining intensity (0, none; 1, weak; 2, moderate; 3, strong) multiplied by the point of the percentage of stained cells (0: positive cells = 0; 1: positive cells ≤25%; 2: 26%–50%; 3: 51%–75%; 4: ≥75%). The range of this calculation was 0–12.

The expressions of ER by immunohistochemistry (IHC) before and after NAC were stable (**Figures 2A, B**) and decreased (**Figure 2C**). The expressions of CLDN6 by IHC before and after NAC in patients were increased, stable, and decreased (**Figures 2A–C**). The expression of ER and CLDN6 with NAC response is shown in **Figure 2D**.

### Retrospective Survival Analysis and Cox Hazard Assessment

**Figure 3:**
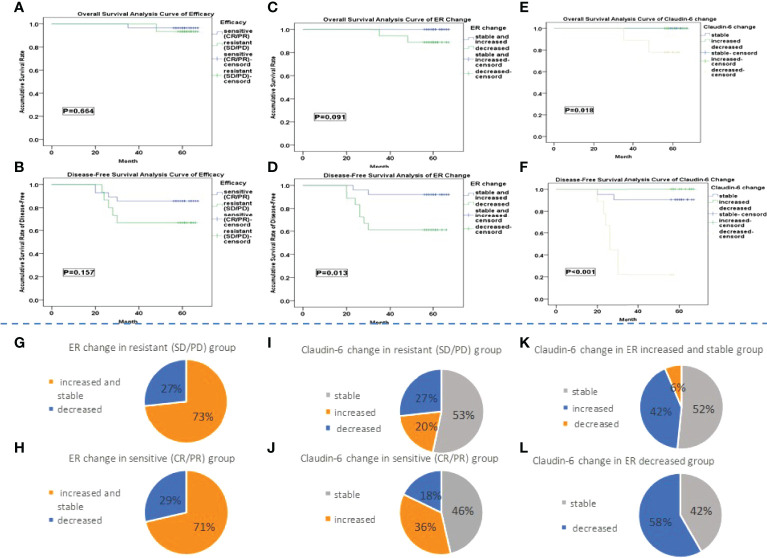
**(A–F)** The Kaplan–Meier survival analysis. **(G, H)** The percentage of patients with different ER change groups in the sensitive and resistant group is shown. **(I, J)** It showed the percentage of patients with different CLDN6 change group in sensitive and resistant group. **(K, L)** The percentage of patients with different CLDN6 change groups in different ER change groups is shown.

There were 28 patients in the sensitive group (CR+PR) and 15 patients in the resistant group (SD+PD). Significant differences were found in cancer diameter, cancer thrombosis, number of positive nodes and total lymph nodes, and endocrine therapy after NAC between two groups (p < 0.05). With a median follow-up time of 61 months, OS was 95.4% in the sensitive group and 93.3% in the resistant group (p = 0.655). There was no significant difference in median OS time (67 months in the sensitive and resistant groups, p = 0.423). DFS was 85.7% in the sensitive group and 66.7% in the resistant group (p = 0.184). There was no significant difference in median DFS time (67 months in the sensitive group and 66 months in the resistant group, p = 0.258) (**Table 1**). Meanwhile, no difference was observed in the curves for OS (p = 0.664) or DSF (p = 0.157) between the two groups (**Figures 3A, B**).

Among the 43 patients, 25 patients were classified into the ER increased and stable group and 18 patients into the ER decreased group. There were significant differences in the number of positive nodes and the ER expression after NAC between two groups (p < 0.05). With a median follow-up time of 61 months, the 5-year OS rate were 100% in the ER increased and stable group and 88.9% in the ER decreased group (p = 0.092). The median OS time was 67 months in both the ER increased and stable group and ER decreased group (p = 0.049). The 5-year DFS rates were 92.0% in the ER increased and stable group and 61.1% in the ER decreased group (p = 0.015). The median DFS times were 67 months in the ER increased and stable group and 66 months in the ER decreased group (p = 0.006) (**Table 1**). No difference was observed in the OS curves (p = 0.091) between two groups (**Figure 3C**), while the difference in the DFS curves was significant (p = 0.013) (**Figure 3D**).

The numbers of patients in the CLDN6 increased, stable, and decreased groups were 13, 21, and 9, respectively (**Table 1**). There were significant differences in number of positive nodes, ER expression, and change among the three groups (p < 0.05). With a median follow-up time of 61 months, the 5-year OS rates were 100% in the CLDN6 increased group and CLDN6 stable group and 77.8% in the CLDN6 decreased group (p = 0.017). The median OS times were 67 months in the CLDN6 increased group and CLDN6 stable group and 63 months in the CLDN6 decreased group (p = 0.007). The 5-year DFS rates in the CLDN6 increased, stable, and decreased groups were 100%, 90.5%, and 22.2%, respectively (p < 0.001). The median DFS times were 67 months in the CLDN6 increased group and CLDN6 stable group and 26.75 months in the CLDN6 decreased group (p < 0.001). Significant differences were observed in OS and DSF surviving curves among the groups (p = 0.018, p < 0.001) (**Figures 3E, F**).

There was no significant difference in the proportion of patients with neither the change of ER (**Figures 3G, H**) nor the change of CLDN6 (**Figures 3I, J**) after NAC between the sensitive and resistant groups. There was a significant difference in the proportion of patients with the different ER change among the CLDN6 increased, stable, and decreased groups (p = 0.012) (**Figures 3K, L**).

There were significant differences in age, histological grade, number of positive nodes, and changes of ER and CLDN6 in cancer progression (all p < 0.05) (**Table 2**).

**Table 2:** The Cox multifactor analysis-related DFS and OS.

Parameters	Tumor progression	
	Sig.	EXP(B)
**Age**	0.023	0.286
**Menopause**	0.947	0.000
**Family history**	0.937	0.000
**Response to NAC**	0.652	0.494
**Histological grade**	0.031	3,137.901
**Number of positive nodes after NAC**	0.047	1.770
**Cancer thrombosis**	0.065	12.421
**ER change**	0.022	1,476,043.874
**Claudin-6 change**	0.024	37.668

## Discussion

It has been reported that multiple endogenous factors contribute to affect intracanceral oxygenation, including hemoglobin, carbon dioxide, distribution of microvessels, and respiratory ventilation function; as an ectogenous factor, NAC has periodic and dynamic effects on intracanceral oxygenation (24). The mean intracanceral oxygenation in the present study demonstrated that cycling hypoxia was induced by NAC, regardless of the cancer responses to NAC (**Figure 1**). Indeed, hypoxia stress has been shown to be a selective pressure for the cancer tissue to induce extracellular matrix remodeling, epithelial–mesenchymal transition, and so on (26). Morphological observations have proved the frequent occurrence of central necrosis due to the lack of effective blood supply (27). Hypoxic selection occurred under conditions of severe cycling hypoxia, leading to treatment resistance (28, 29). We observed a decreasing trend in the mean intracanceral oxygen concentration in the chemotherapy-sensitive group, suggesting that with the shrinking of cancer, the hypoxic area inside the cancer was gradually increasing (**Figure 1K**). This phenomenon was not evident in the resistant group.

Consistent with a previous study, the response to NAC did not predict the prognosis of the patients with LAC (4). We observed that ER expressions increased (few), stabilized, or decreased after NAC in LAC. Survival analysis showed that patients whose ER expression was decreased after NAC had poorer prognosis compared with patients of increased and stable ER expressions (**Figures 3C, D**). It has been reported that ER expression is positively correlated with the benefit of long-term endocrine therapy (30). Therefore, we speculated that the poor prognosis of patients with decreased ER expression in this study may be related to the reduced benefit from endocrine therapy. Interestingly, CLDN6 expression was also changed in NAC. Survival analysis suggested that those with a decreased expression of CLDN6 had a poor prognosis with the majority of cases being in the ER decreased group (**Figures 3E, F, I**). As indicated by Quan, the expression of CLDN6 might be induced by ERα (18). More importantly, NAC induces cancer cycling hypoxia which might be related to the phenotypic drift of cancer; the decrease in ER and CLDN6 could clearly indicate bad prognosis. The COX proportional hazard model of cancer progression suggested that, in addition to age, histological grade, and lymph node status, the change in ER and CLDN6 expression had a significant effect on cancer progression (**Table 1**). Similar to previous studies, age, histological grade, and lymph were important prognostic factors in LABC (31–33).

**Figure 4:**
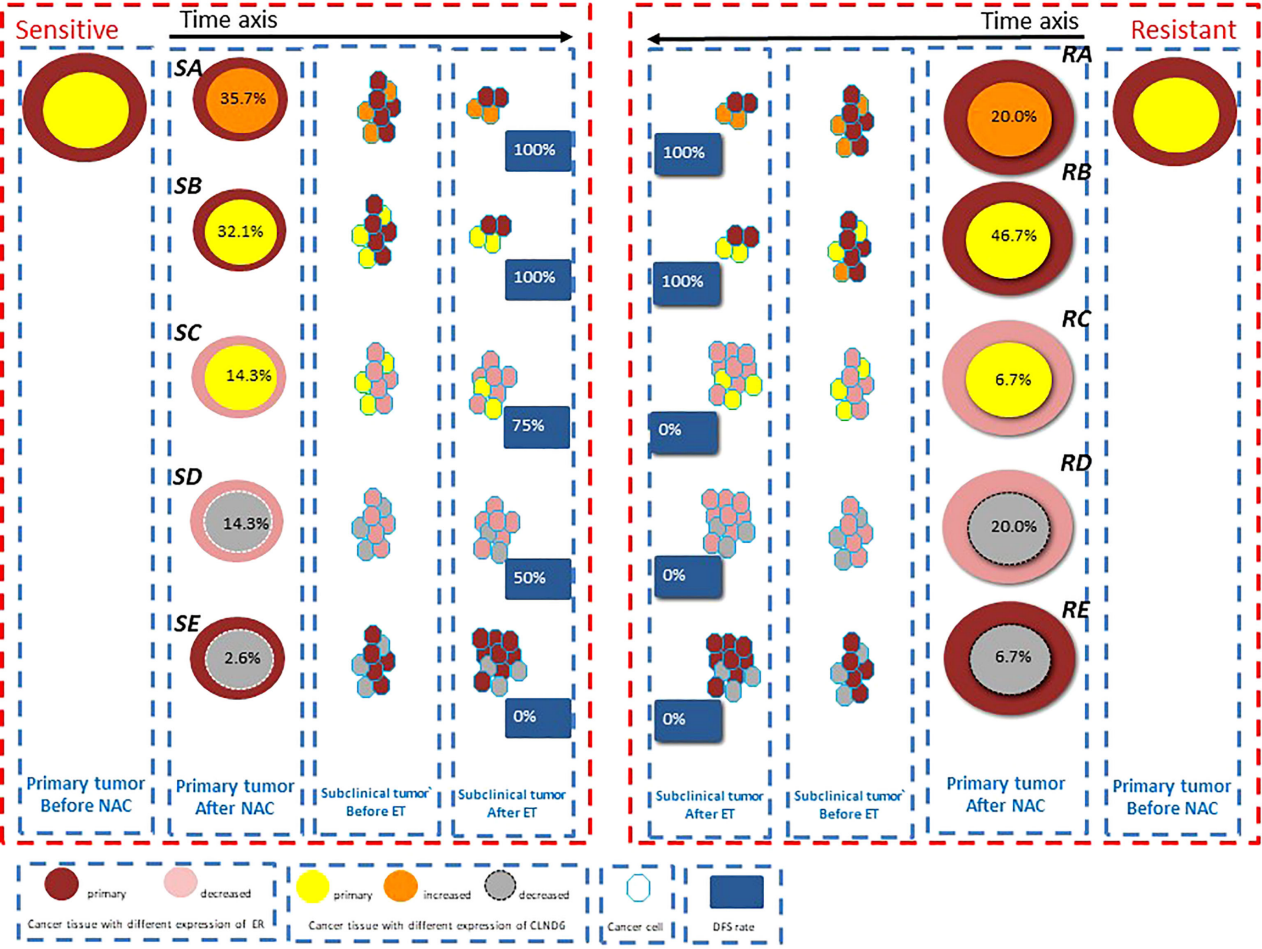
The model hypothesis based on the change of estrogen receptor (ER) and claudin-6 (CLDN6) expression in luminal A cancer (LAC) after neoadjuvant chemotherapy (NAC) is shown to illustrate the relationship between the responses to NAC of LAC (including hypoxia) and long-term prognosis. The different sizes of the circle show its responses to NAC. The percentage in the circle shows the proportion of patients with this possibility in the sensitive or resistant group. The percentage in the blue block shows the DFS rate of the patients with this possibility.

We established a hypothesis based on the change in ER and CLDN6 expression in LAC after NAC to illustrate the relationship between the responses to NAC of LAC and the long-term prognosis. The hypoxia area could be shrinking or stable in both sensitive and resistant groups after NAC. As shown in **Figure 4**, LAC could be sensitive or resistant to NAC. Two trends in the change in ER expression (increased and stable vs. decreased) and three trends in the CLDN6 expression change (increased, stable, and decreased) were observed after NAC. In the theory, we speculated that there were 24 possibilities for LAC after NAC. All the cases with shrinking hypoxic area occurred in the sensitive group; all the cases with stable hypoxic area occurred in the resistant group. Meanwhile, no single case was found in the sensitive or resistant group which demonstrated a decreased ER expression with increased CLDN6 expression. In fact, we only obtained the following 10 possibilities (**Figure 4**). Furthermore, the proportion of increased or stable ER and CLDN6 expression after NAC was similar and about 67% in the sensitive and resistant groups. Patients in this situation would have good prognosis, and with a median follow-up time of 61 months, the DFS rate was 100% (**Figure 4**, SA, SB, RA, RB). It was similar with previous studies that the high expression level of ER after NAC might predict a good long-term prognosis from the benefit of endocrine therapy (30, 34). According to our study, the good prognosis in these patients seemed not related to the efficacy of NAC but related to the increased or stable expression of ER and CLDN6 after NAC. Moreover, the decreasing level of ER expression after NAC might influence the benefit level of endocrine therapy (35). Furthermore, the decreasing level of CLDN6 expression after NAC was an important predictor for poor prognosis, regardless of the change in ER expression after NAC (**Figure 4**, SD, SE, RD, RE). At the same time, the poor prognosis of these patients with decreased CLDN6 expression after NAC was not related to the efficacy of NAC. In addition, the patients with decreased ER and stable CLDN6 expressions had good prognosis in the NAC-sensitive group and poor prognosis in the NAC-resistant group (75% vs. 0%). With the situation of patients with Her2 overexpression or TNBC, the good effect of NAC could transform to good prognosis (3). However, there were only five patients with decreased ER and stable CLDN6 expressions (four in the sensitive group and one in the resistant group), and further research is required to verify the hypothesis. In addition, the change in the level of intracanceral cycling hypoxia represented the proportional relationship between the cancer tissue size and the hypoxia area, and the change of intracanceral cycling hypoxia was related to the efficacy of NAC, but not to the prognosis. In fact, cycling hypoxia is one of the evolutionary dynamics to induce intracanceral heterogeneity (36). It was for the heterogeneous subclone cells to survive from cycling hypoxia which could be distinguished by the expression levels of ER and CLDN6. However, the final expression levels of ER and CLDN6 after NAC were not related to the cycling hypoxia, and the expression changes in ER and CLDN6 after NAC determined the long-term prognosis.

In general, intracanceral cycling hypoxia could be induced by NAC in LAC, and the pattern of cycling hypoxia might relate to the response of NAC, but not to prognosis. For most of the patients with local advanced LAC, the long-term prognosis had no connection with the response to NAC: about 67% patients would maintain increased or stable ER and CLDN6 expression, benefit from endocrine therapy, and have good prognosis, and about 20% patients with decreasing expression of CLDN6 would undergo the poor prognosis. Besides, only 13% patients’ prognosis was related to the response of NAC, and the patients maintained a stable CLDN6 expression and decreased ER expression. Anyway, the expression changes of ER and CLDN6 after NAC would be an important marker to predict long-term prognosis for the patients with local advanced LAC. Further studies are required to research the relationship among cycling hypoxia, CLDN6, and resistance of endocrine therapy, and prospective trials with a large sample should be carried out to verify the clinical significance of markers. The main limitation of the study was the sample size. A larger sample would have better represented the population we studied. However, we believe that our findings may provide valuable guidance for clinicians in their decision-making in managing patients with luminal-type LABC and lay a foundation in future research in LABC patients undergoing NAC. In the future study, we will increase the sample size and perform puncture again 2 weeks after the first cycle of NAC to determine the effect of NAC on ER and CLDN6 expressions and predict the long-term prognosis of patients undergoing NAC.

## Conclusion

The changes in CLDN6 and ER after NAC might be important prognostic factors in local advanced LAC, and the phenotypic drift is closely related to the cycling intracanceral hypoxia induced by NAC.

## Data Availability Statement

The original contributions presented in the study are included in the article/supplementary material. Further inquiries can be directed to the corresponding author.

## Ethics Statement

The studies involving human participants were reviewed and approved by the Ethics Committee of Shengjing Hospital of China Medical University. The patients/participants provided their written informed consent to participate in this study.

## Author Contributions

YL and JL designed the proposal and drafted the manuscript. YK participated in the immunohistochemical stain and analysis. YM and JZ carried out the ultrasound analysis. SJ, QS, ZW, YC, and YS participated in the data collection. YZ, SJ, YC, YS, YL, and JL participated in the total data analysis. All authors contributed to the article and approved the submitted version.

## Conflict of Interest

The authors declare that the research was conducted in the absence of any commercial or financial relationships that could be construed as a potential conflict of interest.

## Publisher’s Note

All claims expressed in this article are solely those of the authors and do not necessarily represent those of their affiliated organizations, or those of the publisher, the editors and the reviewers. Any product that may be evaluated in this article, or claim that may be made by its manufacturer, is not guaranteed or endorsed by the publisher.
